# Metabolic syndrome in urban city of North-Western Nigeria: prevalence and determinants

**DOI:** 10.11604/pamj.2016.23.19.5806

**Published:** 2016-01-27

**Authors:** Anas Ahmad Sabir, Abdulgafar Jimoh, Sandra Omozehio Iwuala, Simeon Alabi Isezuo, Lawal Suleiman Bilbis, Kaoje Umar Aminu, Sani Atta Abubakar, Yusuf Saidu

**Affiliations:** 1 Department of Medicine, Usmanu Danfodiyo University Teaching Hospital Sokoto, Nigeria; 2 Pharmacology Department Usmanu Danfodiyo University Sokoto, Nigeria; 3 Department of Medicine, Lagos University Teaching Hospital, Nigeria; 4 Department of Biochemistry, Usmanu Danfodiyo University, Sokoto, Nigeria; 5 Department of Community Health, Usmanu Danfodiyo University Teaching Hospital Sokoto, Nigeria; 6 Department of Medicine, Ahmadu Bello University Teaching Hospital, Zaria, Nigeria

**Keywords:** Metabolic syndrome, dyslipidemia, obesity

## Abstract

**Introduction:**

The aim of this study was to investigate the prevalence of metabolic syndrome in Sokoto metropolis of North-Western Nigeria.

**Methods:**

A cross-sectional community based study was carried out. Four hundred and ten subjects (201 males and 209 females) were recruited for the study using a multi-stage sampling technique. Demographic and the life style data was obtained from the participants. Evaluation of anthropometric variables, fasting blood sugar, lipid profiles, insulin resistance and blood pressure was performed. The classification of metabolic syndrome was based on the NCEP ATP III guidelines.

**Results:**

The mean (SD) age of the sample population was 39.6 (14.4) years. The mean (SD) age of the male subjects was 38.4(14.9) years and that of the females was 40.8(13.9) years (p> 0.05). The overall prevalence of metabolic syndrome was 35.1% with the females having 42.83% and the males 27.36%. The frequencies of metabolic syndrome parameters in the study subjects were low HDL (56.1%), hypertension (46.1%), dysglycemia (32.7%), central obesity (28%), and elevated triglycerides (22.4%). Most of the women had low HDL (62.2%) and central obesity elevated (49.8%).

**Conclusion:**

Metabolic syndrome is common in residents of North-Western Nigeria, commoner in the females than males. Risk factors for metabolic syndrome should be detected in normal individuals for implementing effective preventive measures.

## Introduction

Metabolic syndrome is a cluster of metabolically related cardiovascular risk factors, the core components of which comprise of central obesity, insulin resistance, dyslipidaemia and hypertension [1]. The presence of the metabolic syndrome predicts the risk of cardiovascular disease in both patients with diabetes mellitus as well as in those without diabetes mellitus [2, 3]. Central to development of the metabolic syndrome appears to be the presence of increased insulin resistance occasioned by oxidative stress [3, 4]. A large number of any populations with metabolic syndrome will have an important implication for the health sector. Lifestyle factors (nutrition, environment, stress, smoking, alcohol and drug intake, exercise, etc.) have a considerable, but not always well understood impact on a variety of health issues. The prevalence of metabolic syndrome is rising worldwide with urbanization and sedentary lifestyle being risk factors [5, 6]. There is paucity of data on the prevalence of metabolic syndrome among the Hausa-Fulani ethnic group of Sokoto, North-Western Nigeria. The Hausa-Fulani usually have a lean physique that should prevent against metabolic syndrome. However, with modernization some have become obese and adopted sedentary lifestyles that are risk factors for metabolic syndrome [7]. The objective of this study is to investigate the prevalence and determinants of metabolic syndrome in an urban community of Sokoto state, Nigeria.

## Methods

**Study Location:** the study was conducted in Sokoto metropolis in the Sudan savannah zone of North western Nigeria. The state is bordered to the north by the republic of Niger and to the east and southwest respectively by Zamfara and Kebbi states of Nigeria. The state had a population of 3.69 million according to the 2006 census figures over 90% of whom are Muslim Fulani and Hausas [8].

**Participants:** consenting adults (above 18 years of age) were recruited. Trained research assistants administered questionnaires and obtained the measurements including collection of blood samples.

**Ethical consideration:** besides obtaining permission and consent from local authorities and individuals respectively, the study protocol was approved by the Research and Ethics Committee of Sokoto state, Nigeria. Study Design: a cross-sectional community based study was carried out. Four hundred and ten subjects (201 males and 209 females) were recruited for the study. Using a multi-stage sampling technique, three districts of Gidan Dare, Gidan Igwai and Arkilla were selected. The first stage involved random sampling selection of some districts; while the second stage involved selection of some households using clustered sampling technique from the districts selected. Pretested questionnaire was administered by trained research assistants. Demographic and the life style data was obtained from the participants. Evaluation of anthropometric variables, fasting blood sugar, lipid profiles, insulin resistance and blood pressure was performed.

**Operational definitions:** the classification of metabolic syndrome was based on the National Cholesterol Education Program-Adult Treatment Panel (NCEP ATP III) guidelines (Table 1) [1]. Metabolic syndrome was diagnosed when any three features of table 1 were present. Data Management and Statistical Analysis: raw data was entered into a spreadsheet (Microsoft Excel 2007) before exporting to Epi-Info version 3.3.2 for analysis. Significance of differences between group means was assessed using Student's t-test while chi-squared statistic was employed to determine significance of results of comparison of proportions between groups. Linear relationships were determined using Pearson's correlation coefficients (r). The level of statistical significance is set at p < 0.05.

**Table 1:** ATP III Clinical identification of metabolic syndrome

Risk Factor	Defining level
Abdominal obesity (waist circumference)	
Men	>102cm
Women	>88cm
Triglycerides	≥150mg/dl
HDL Cholesterol:	
Men	<40mg/dl
Women	<50mg/dl
Blood pressure	≥135/ ≥ 85 mmHg
Fasting glucose	≥100mg/dl

## Results

**Socio-demographic charactheristic:** the mean (SD) age of the sample population was 39.6 (14.4) years. The mean (SD) age of the male subjects was 38.4(14.9) years and that of the females was 40.8(13.9) years (p> 0.05).

**Anthropometric and blood pressure measurements:** the anthropometric and blood pressure values of the research participants are shown in Table 2. The females had higher body mass index (BMI) and waist circumference (WC) than the males.

**Table 2:** Anthropometric and blood pressure values of the research participants

Variable	All (n=410)	Male (n=201)	Female (n=209)	p value
Weight (Kg)	62.9 (14.1)	65.2 (12.9)	61.1 (14.7)	0.050
Height (cm)	163.6 (60.8)	163.3 (60)	159.5 (54.2)	0.001
BMI (kg/m^2^)	23.1 (5.5)	22.6 (4.9)	23.6 (5.9)	0.197
WC (cm)	86.2 (13.7)	80.9 (13.1)	90.8 (12.6)	0.001
SBP (mmHg)	138.6 (26.6)	138.6 (19.7)	138.5 (31.6)	0.970
DBP (mmHg)	79.3 (16.4)	79.9 (15.1)	78.8 (17.5)	0.65

BMI, Body Mass Index; DBP, diastolic blood pressure; SBP, systolic blood pressure; WC, waist circumference; p value, significance of difference between male group and female group

**Figure 1:**
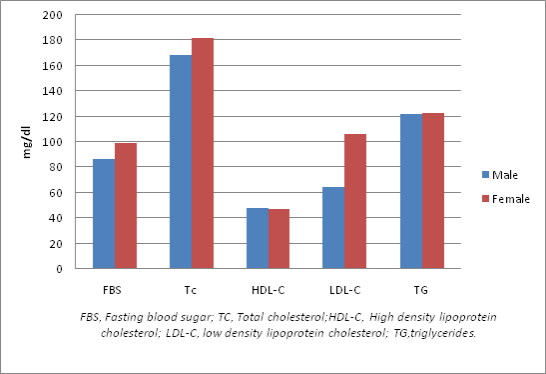
Metabolic profile of the research participant

**Metabolic profile:** the metabolic profile of the research participants is shown in Figure 1. The females had significantly higher low density lipoprotein cholesterol (LDL-C) than the males (p=0.001). The total cholesterol (TC) is also higher in the females but not statistically significant (p=0.065) as is shown in Table 3.

**Table 3:** Metabolic profile of the research participants

Variable	All (n=410)	Male (n=201)	Female (n=209)	p value
FBS(mg/dl)	91.8 (25.2)	86.4 (16.2)	99 (32.4)	0.17
TC (mg/dl)	175.7 (50.7)	168.2 (58)	182.2 (42.7)	0.065
HDL-C (mg/dl)	47.5 (22.5)	48.0 (15.9)	47.1 (17.7)	0.847
LDL-C (mg/dl)	86.7 (48.5)	64.3 (32.7)	106.2 (51.1)	0.001
TG (mg/dl)	122.4 (64.6)	121.9 (65.4)	122.8 (58.6)	0.924

FBS, Fasting blood sugar; TC, Total cholesterol;HDL-C, High density lipoprotein cholesterol; LDL-C, low density lipoprotein cholesterol; TG,triglycerides; n, number; mg/dl, milligrams per deciliter; n, number; p value, significance of difference between male group and female group

**Determinants of metabolic syndrome:** the determinants of metabolic syndrome are as shown in Table 4. Decreased high density lipoprotein cholesterol (HDL) and hypertension were the most common components of metabolic syndrome respectively. Most of the women had low HDL (62.2%) and central obesity elevated (49.8%) also. Increased waist circumference, plasma glucose, triglycerides and decreased HDL were commoner in the female than the male subjects.

**Table 4:** Determinants of Metabolic Syndrome according to gender

Determinant	Number (%)			p value
	All (n=410)	Female (n=209)	Male (n=201)	
↑WC	115 (28.0)	104 (49.8)	11 (5.5)	0.001
Hypertension	189 (46.1)	93 (44.5)	96 (47.7)	0.897
Dysglycemia	134 (32.7)	72 (34.4)	62 (30.8)	0.360
↑TG	92 (22.4)	52 (24.9)	40 (19.9)	0.416
↓HDL	230 (56.1)	130 (62.2)	100 (49.8)	0.683

↑WC= increased waist circumference, ↑TG== increased triglycerides, ↓HDL= decreased high density lipoprotein; *p value, significance of difference between male group and female group*

**Figure 2:**
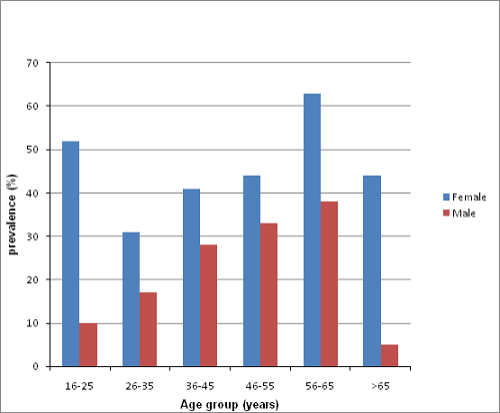
Prevalence of metabolic syndrome by sex and age group

**Prevalence of metabolic syndrome:** the distribution of metabolic syndrome by age group and gender is shown in Figure 2. The overall prevalence of metabolic syndrome was 35.1% with the females having 42.83% and the males 27.36%.

## Discussion

The 35.1% prevalence of metabolic syndrome found in this study is high. This is contrary to previous reports of low prevalence of metabolic syndrome in Africa [9, 10]. The high prevalence may be because the research was conducted in the urban area. Urbanization is known to be associated with physical inactivity as well as nutritional transition to refined, low fibre, calorie dense meals [11, 12]. Similar findings were obtained by other researchers in other part of the world. Akintunde et al [13] found prevalence 35% among hypertensives in Nigerians. Gupta et al [14] found the prevalence of metabolic syndrome in an Indian urban population to be 31.6%. Sarkar et al [15] also found significantly higher prevalence of metabolic syndrome in the urban than the rural subjects (37% vs. 4%, p < 0.05) in India. The prevalence is however lower than 86% Ogbera [16] found in an Urban community in Lagos Nigeria, although the study was hospital based and involved only patients with diabetes mellitus already. Kaler et al [17] found upto 52% prevalence of metabolic syndrome in Western Canada which is more industrialized area than our study area. The prevalence of metabolic syndrome was higher in the females than the males. This has been found in several other studies [18–20]. The difference might be because of the different cut off values used for the definition of obesity and dyslipidaemia in the different sex groups. Additionally, previous studies in the same population found females to be more obese and are less engaged in physical activities than males [7]. There was difference in the rates of appearance of the components of metabolic syndrome. We found hypertension and low HDL cholesterol to be the most prevalent components of the metabolic syndrome. However, central obesity was also found to be very common in the females (49%). Similar finding of central obesity being more common in females was obtained by other researchers. Beigh et al [21] found the most the most common component of metabolic syndrome to be hypertension (38%), low HDL (36%) and central obesity (29%). Similarly, Isezuo et al [20] also found hypertension and obesity to be common components of metabolic syndrome but with less common low HDL-C although the study was among patients with diabetes mellitus only.

Limitation of the study: the study was cross-sectional hence causal relationship cannot be established.

## Conclusion

Metabolic syndrome is very common in residents of North-western Nigeria, commoner in the females than males. There is an urgent need for mass public health education so as to reduce the potential complications of the risk factors for metabolic syndrome. Additionally risk factors for metabolic syndrome should be detected in normal individuals for implementing effective preventive measures.

### What is known about this topic

- The prevalence of metabolic syndrome is rising worldwide with urbanization and sedentary lifestyle being risk factors
- Metabolic syndrome is a cluster of metabolically related cardiovascular risk factors, the core components of which comprise of central obesity, insulin resistance, dyslipidaemia and hypertension

### What this study adds

- We found hypertension and low HDL cholesterol to be the most prevalent components of the metabolic syndrome
- We found Metabolic syndrome to be very common in residents of North-western Nigeria, commoner in the females than males
